# Molecular heterogeneity of guanine nucleotide binding-protein γ subunit 4 in left- and right-sided colon cancer

**DOI:** 10.3892/ol.2021.12488

**Published:** 2021-01-26

**Authors:** Jintian Song, Jianwei Yang, Rongbo Lin, Xiongchao Cai, Liang Zheng, Yigui Chen

Oncol Lett 16: Article no. 34, 2020; DOI: 10.3892/ol.2020.12197

Subsequently to the publication of this article, the authors have realized that the affiliation was printed incorrectly: The institute was incorrectly printed as “The Affiliated Cancer Hospital of Fujian Medical University”, whereas only the name “Fujian Medical University Cancer Hospital” is approved by the authors’ hospital. Therefore, the authors’ affiliation information should have appeared as follows (changes are highlighted in bold):

JINTIAN SONG, JIANWEI YANG, RONGBO LIN, XIONGCHAO CAI, LIANG ZHENG and YIGUI CHEN

Department of Abdominal Oncology, **Fujian Medical University Cancer Hospital**, Fuzhou, Fujian 350014, P.R. China

The authors apologize for this oversight on their part, and for any inconvenience caused.

